# Complete Genome Sequence of Acidithiobacillus ferridurans JAGS, Isolated from Acidic Mine Drainage

**DOI:** 10.1128/MRA.00033-20

**Published:** 2020-03-26

**Authors:** Jinjin Chen, Yilan Liu, Patrick Diep, Andrew Jo, Camilla Nesbø, Elizabeth Edwards, Vladimiros Papangelakis, Radhakrishnan Mahadevan

**Affiliations:** a Department of Chemical Engineering and Applied Chemistry, University of Toronto, Toronto, Canada; b Department of Biological Sciences, University of Alberta Edmonton, Alberta, Canada; c Institute of Biomaterials and Biomedical Engineering, University of Toronto, Toronto, Canada; University of Southern California

## Abstract

We report a complete genome sequence of Acidithiobacillus ferridurans JAGS, determined using PacBio single-molecule real-time (SMRT) sequencing. The circular genome of JAGS (2,933,811 bp; GC content, 58.57%) contains 3,001 protein-coding sequences, 46 tRNAs, and 6 rRNAs. Predicted genes indicate the potential to fix CO_2_ and N_2_ and to utilize Fe^2+^, S^0^, and H_2_ as energy sources.

## ANNOUNCEMENT

Harnessing biomining microorganisms to process mine wastes is increasing in popularity. In particular, the genus *Acidithiobacillus* is of importance because of its adaptation to extremely acidic and heavy metal-rich environments (1). Acidithiobacillus ferridurans is a newly reported species with high acid tolerance and superior growth on hydrogen, compared to other *Acidithiobacillus* species (2). However, only one complete genome sequence, that of A. ferridurans JCM 18981, is available (3), which prompted us to isolate and sequence the genome of A. ferridurans JAGS to better understand the genetic properties of this species.

In this article, we report the complete genome sequence of A. ferridurans JAGS, which was isolated from an acidic mine drainage (AMD) sample collected in September 2010 from Clarabelle Mill, near Sudbury, Ontario, Canada. Govindarajan (4) enriched this AMD culture and found one dominant species, which belonged to the *Acidithiobacillus* genus and made up 92.6% of the enriched culture, based on 16S rRNA gene sequence analysis. The strain was purified by streaking a single colony three consecutive times on *Thiobacillus* solid medium 1 (TSM1) and then was cultured in 9K liquid medium (5). Genomic DNA was extracted using the GeneJet genomic DNA preparation kit (Thermo Scientific, USA). A DNA library was prepared by following the Pacific Biosciences protocol for preparing multiplexed microbial libraries using SMRTbell Express template preparation kit 2.0 and was sequenced on the PacBio Sequel system at the Genome Québec facility (Montréal, QC, Canada) (6). SMRTLink v.6.0.0 was used to filter and assemble the subreads with HGAP4, and Circlator v.1.4.1 was used for circulation, as described for the PacBio pipeline (7). A total of 145,583 filtered reads were obtained, with an *N*_50_ value of 8,294 bp, a base count of 979,131,206 bp, and coverage of 333-fold. The preassembled reads numbered 6,114, with an *N*_50_ value of 14,365 bp and a base count of 73,033,378 bp. A. ferridurans JCM 18981 was used as a reference for the assembly. NCBI PGAP was used for genome annotation (8). A BLAST search (https://blast.ncbi.nlm.nih.gov/Blast.cgi) of the predicted 16S rRNA gene of strain JAGS was performed. The average nucleotide identity based on BLAST (ANIb) was calculated using JSpeciesWS (9). Default parameters were used for all software, unless otherwise specified.

The complete genome sequence of strain JAGS contains 2,933,811 bp, with a GC content of 58.57%. No plasmid was detected in this strain. Genome analysis predicted a total of 3,001 protein-coding sequences (CDSs), 2,833 proteins, 46 tRNAs, and 6 rRNAs. The functional annotation of the CDSs indicated the potential to use ferrous iron, reduced sulfur compounds, and hydrogen as energy resources and to fix CO_2_ and N_2_ as carbon and nitrogen sources, respectively. JAGS was identified as a member of A. ferridurans, based on its 100% identity of 16S rRNA genes and 98.65% ANIb value, compared with A. ferridurans JCM 18981. The genome sequence of A. ferridurans JAGS may provide a valuable resource for better understanding the evolutionary relationship between A. ferridurans and A. ferrooxidans, as well as their mechanisms of adaptation to challenging environments.

### Data availability.

The complete genome sequence has been deposited in GenBank under accession number CP044411, BioProject accession number PRJNA573091, BioSample accession number SAMN12799170, and SRA accession number SRR10222673.
